# Non-traditional lipid profiles associated with ischemic stroke not hemorrhagic stroke in hypertensive patients: results from an 8.4 years follow-up study

**DOI:** 10.1186/s12944-019-0958-y

**Published:** 2019-01-08

**Authors:** Jia Zheng, Zhaoqing Sun, Xingang Zhang, Zhao Li, Xiaofan Guo, Yanxia Xie, Yingxian Sun, Liqiang Zheng

**Affiliations:** 1 Department of Clinical Epidemiology, Library, Department of Health Policy and Hospital Management, Shengjing Hospital of China Medical University, Shenyang, 110004 People’s Republic of China; 2 Department of Cardiology, Shengjing Hospital of China Medical University, Shenyang, 110004 People’s Republic of China; 3 Department of Cardiology, First Affiliated Hospital of China Medical University, Shenyang, 110001 People’s Republic of China

**Keywords:** Ischemic stroke, Hemorrhagic stroke, Traditional lipids, Non-traditional lipids, Prospective study

## Abstract

**Background:**

Studies have shown that non-traditional lipid profiles have a better association with stroke than traditional blood lipids in clinical applications, other studies have drawn different conclusions.

**Methods:**

This study was a large-scale study with a median follow-up of 8.4 years. The hazard ratio (HR) and 95% Confidence interval (CI) of lipid variables for risk of incident stroke were analyzed by multivariable Cox proportional hazard models.

**Results:**

During the follow-up, 502 new strokes (310 ischemic, 187 hemorrhagic, and 5 unclassified strokes) occurred among the 5099 hypertensive patients. Comparing with the lowest quarter, the HR of future ischemic stroke (IS) in the highest were 1.41(95%CI, 1.03–1.92) for TC, 1.60 (95%CI, 1.15–2.22) for TG, 1.03 (95%CI, 0.75–1.42) for HDL-C, 1.77 (95%CI, 1.29–2.44) for LDL-C, 1.42 (95%CI, 1.03–1.94) for non-HDL, 2.09 (95%CI, 1.45–3.00) for TC/HDL, 2.08 (95%CI, 1.46–2.96) for LDL/HDL, 1.86 (95%CI 1.33–2.60) for TG/HDL, respectively. No significant association was observed between lipid-related indicators and hemorrhagic stroke. The results of statistical differences showed that the correlation between LDL/HDL and the risk of ischemic stroke in non-traditional lipids was higher than that of other traditional lipids (*P* < 0.001), except for LDL (*P* = 0.056).

**Conclusions:**

We didn’t find that HDL was associated with the risk of stroke and all the lipid parameters were not associated with the risk of hemorrhagic stroke. LDL/HDL was associated with a higher risk of ischemic stroke than other lipids and should be considered for clinical diagnosis and future disease prevention.

## Background

According to the Global Burden of Disease (GBD) study, stroke has become the main cause of global disability and brings a heavy economic burden to society [1]. At the same time, hypertension has been considered as the second main risk factor of disability-adjusted life-years and deaths [2]. More than one-quarter of the Chinese adult population had hypertension [3]. Hypertensive patients have higher risk in the matter of stroke status than that in subjects with normal blood pressure [4]. Therefore, investigating the strong predictors of stroke for hypertensive patients is very important.

Currently, it is an active research area that lipids profiles, including traditional and non-traditional lipids profiles, have been confirmed to be independent predictors for CVD in different patients [5–7]. Some studies showed that low-density lipoprotein cholesterol (LDL-C), non-high-density lipoprotein cholesterol (non-HDL-C), and TC/HDL-C were powerful in the aspect of prediction for CVD [6–9]. Owing to better reflection of the associations between lipid profiles, LDL-C/HDL-C was considered as more useful indicator than isolated lipid values for CVD risk assessment. [10, 11]. Additionally, some study indicates that non-HDL-C is a better indicator for the development of vascular disease than LDL-C [6]. But lipids profiles as a major indicator for the prevention of stroke still face considerable uncertainty [12–20]. For instance, Tirschwell DL et al. found that increased cholesterol levels and reduced high-density lipoprotein (HDL) levels were associated with an increased risk of IS [16]. Shahar E et al. [19] found that blood cholesterol levels were not related to stroke as well as coronary heart disease (CHD). Bowman T S et al. [21] found TC, HDL, TG were not significantly associated with IS risk after adjustment with participants of Physicians’ Health Study (PHS). Zhang Y et al. [22] found a positive association between TC/HDL-C and all stroke and IS but not hemorrhagic stroke.

Therefore, the role of lipids profiles at the aspect of stroke status and risk assessment also needs to further discuss. At the same time, it is lacking in the aspect of using large-scale prospective cohort study to verify the role of lipids profiles of prediction for stroke, especially in Chinese hypertensive patients. Simultaneously, few studies compared the power of traditional and non-traditional lipids indicators in predicting the risk of stroke in hypertensive patients. In order to analyze the above issues, our study investigated the association between stroke and the traditional or non-traditional lipids profiles in large-scale prospective hypertensive patients.

## Methods

### Study population and study design

This was a large-scale, prospective study and was conducted for the first time in 50 rural communities in Fuxin, Liaoning Province, China, from October 2004 to June 2006 using a multistage cluster random sampling design. Six thousand four hundred twelve hypertensive people aged 35 or older were included at the beginning of the study. All of the eligible hypertensive patients were invited to conduct 3 follow-ups with a median follow-up time of 8.4 years (from January to June 2008, from July to October 2010, and from August to October 2014). Of those, 238 people were lost to follow-up because of missing contact information or refusing to accept the follow-up. Six thousand one hundred seventy-four patients completed at least one follow-up visit. After removing baseline patients with coronary artery disease (CAD, concluded angina pectoris, myocardial infarction, arrhythmia) (*n* = 570) and previous stroke (*n* = 507), 5099 hypertensive patients without cardiovascular disease (CVD) were selected for the prospective cohort study. The Ethics Committee of China Medical University has approved the research plan and written informed consent has been formally obtained from all patients or their guardians.

### Stroke assessment

Stroke events were confirmed according to the WHO Multinational Monitoring of Trends and Determinants in Cardiovascular Disease (MONICA) criteria: cases with significant non-vascular etiologic events, including local or global brain disorders that lasted longer than 24 h, but it contained stroke events that had a duration of fewer than 24 h due to death or surgery [23]. This study focused on endpoint events including IS and hemorrhagic stroke. An IS event was a stroke event diagnosed with thrombosis or embolism. Hemorrhagic Stroke including intracerebral hemorrhage stroke (ICH) and subarachnoid hemorrhage stroke (SAH). Transient ischemic attacks (TIA) and silent brain infarctions (cases without clinical symptoms or signs) were not included, neither were events associated with trauma, hematologic disorders, or malignancy. All materials were independently reviewed by the end-point assessment committee, whose members were all blinded to the study participants’ baseline risk factor information. The procedures for obtaining medical records and diagnosing diseases have been described in detail elsewhere [24, 25].

### Blood lipid and serum glucose measurement

The content of blood lipid measurement in this study has been described in detail elsewhere [17, 18]. Before blood collection, we asked patients to fast for at least 12 h. Serum glucose and blood lipids analyses were performed at a certified, central laboratory with an Olympus AU640 autoanalyzer (Olympus, Kobe, Japan). Routine lipids measurements included: TC, TG, HDL-C, and LDL-C. Further calculations of non-traditional indicators included: TC/HDL-C, TG/HDL-C, LDL-C/HDL-C,and Non-HDL-C which was calculated by subtracting HDL-C from TC. Diabetes mellitus was defined as fasting serum glucose levels at least 7.0 mmol/l or plasma glucose concentration of at least 11.0 mmol/l2 h after a 75-g oral glucose load or current treatment with insulin or oral hypoglycemic agents.

### Blood pressure measurement

The blood pressure (BP) measuring device was an electronic blood pressure monitor that had been verified by the British Hypertension Society protocol (Omron; Dalian, Liaoning, China). We measured the blood pressure three times in the same position on the left arm of the subject in the sit-in for more than 5 min and calculated the average of three systolic blood pressure (SBP) and diastolic blood pressure (DBP) to determine the subject’s examination report. High blood pressure was defined as average SBP ≥140 mmHg or/and average DBP ≥90 mmHg or/and use of antihypertensive medications within the previous 2 weeks.

### Collection and definition of other risk factors

Information on data collection and physical examination have been described in detail in the previously published literature [24, 26]. We used face-to-face interviews between patients and doctors who had undergone formal training to obtain lifestyle-related factors (current smoking, current drinking, and different classes of antihypertensive medications). Current smoking was defined as smoking at least one cigarette per day and lasting for at least one year. We converted the different varieties of wine already on the market into the corresponding grams of alcohol at different concentrations. Heavy drinking was defined as more than 1 drink/day for women and more than 2 drinks/day for men during the last year [27].

### Statistical analysis

The categorical variables were described using percentiles. The mean and standard deviation were used to describe the continuous variables. The median (inter-quarter range) method was used to describe the central and discrete trends of continuous variables that did not meet the normal distribution. The Spearman correlation coefficient was used to measure the correlation between each lipid evaluation index. Four equal divisions were made based on baseline blood variables. We used the Cox proportional hazards model to calculate the HR values and corresponding 95% confidence intervals by comparing the second, third, and fourth quartiles with the lowest quartile. Multivariable models were adjusted by age, sex, ethnicity, BMI, current smoking, current drinking, diabetes mellitus, SBP, DBP, and anti-hypertensive medications. We tested the trend based on the lipids variable containing the median value for each quintile. Therefore, we tested the difference of *β-*coefficients with z-transformation (mean = 0; standard deviation = 1) to compare the predictive power of individual BP parameters using Fisher Z test (Considering the collinearity among the lipids, all the lipids indicators were separately tested in a Cox proportional hazards model). Data analysis was conducted using SPSS statistical software Version 22.0, and *P* values less than 0.05 were considered to be statistically significant.

## Results

The baseline mean age (SD) of the 5097 initially no-stroke hypertensive patients in this study was 56.3 (11.2) years, with 56.2% women. Baseline information has been described in detail in Table 1. Correlation analysis found that TC, LDL-C, HDL-C, TG, non-HDL, TC/HDL, TG/HDL, and LDL/HDL were all significantly related (Table 2) (all *P* < 0.05).

**Table 1:** Baseline Characteristics of hypertensive patients

Variables	Summary Values
n	5097
Age, years, Mean (SD)	56.3 (11.2)
Women, n (%)	2866 (56.2)
Han ethnicity, n (%)	4077 (80.0)
BMI, kg/m2,Mean (SD)	23.9 (3.4)
Current smoking, n (%)	2045 (40.1)
Current drinking, n (%)	1503 (29.5)
Taking anti-hypertensive drugs, n (%)	1191 (23.4)
HR, beats/min, Mean (SD)	75.9 (11.1)
Diabetes mellitus, n (%)	401 (7.9)
Lipid,mmol/L, Median (IQR)	
TC	5.17 (4.52–5.84)
TG	1.32 (0.93–1.98)
HDL-C	1.40 (1.20–1.62)
LDL-C	2.73 (2.29–3.22)
BP Parameters, mmHg, Mean (SD)	
SBP	159.7 (21.0)
DBP	94.0 (12.0)
MAP	115.9 (12.7)
PP	65.8 (19.3)

The body mass index (BMI) is the weight in kilograms divided by the square of the height in meters. *BP* blood pressure, *HR* heart rate, *BMI* body mass index, *IQR* interquarter range, *TC* Total cholesterol, *TG* Triglycerides, *HDL-C* high-density lipoprotein cholesterol, *LDL-C* low-density lipoprotein cholesterol. *SBP* Systolic blood pressure, *DBP* diastolic blood pressure, *PP* pulse pressure, *MAP* mean arterial pressure

**Table 2:** Correlation Coefficient between lipid variables

Lipid variables	TC	TG	HDL-C	LDL-C	Non-HDL	TC/HDL-C	TG/HDL-C	LDL/HDL-C
TC	1.00	0.33**	0.73**	0.89**	0.96**	0.13**	0.03**	0.29**
TG	0.33**	1.00	0.08**	0.38**	0.38**	0.26**	0.91**	0.37**
HDL-C	0.73**	0.08**	1.00	0.61**	0.53**	−0.49**	−0.30**	−0.24**
LDL-C	0.89**	0.38**	0.61**	1.00	0.88**	0.20**	0.12**	0.56**
Non-HDL	0.96**	0.38**	0.53**	0.88**	1.00	0.35**	0.15**	0.46**
TC/HDL-C	0.13**	0.26**	−0.49**	0.20**	0.35**	1.00	0.44**	0.77**
TG/HDL-C	0.03**	0.91**	−0.30**	0.12**	0.15**	0.44**	1.00	0.45**
LDL/HDL-C	0.29**	0.37**	−0.24**	0.56**	0.46**	0.46**	0.45**	1.00

**. Correlation was significant at the 0.01 level (2-tailed)

*TC* Total cholesterol, *TG* Triglycerides, *HDL-C* high-density lipoprotein cholesterol, *LDL-C* low-density lipoprotein cholesterol

During the follow-up period, a total of 501 new strokes occurred at the end of the last follow-up, including 310 cases of ischemic stroke, 186 cases of hemorrhagic stroke, and 5 cases of unclassified stroke. In the hypertensive patients, the incidence density of all stroke was 1236.30 per 100,000 person-years (95%CI: 1167.81 per 100,000 person-years - 1304.79 per 100,000 person-years), IS was 764.97 per 100,000 person-years (95%CI: 710.97 per 100,000 person-years - 818.97 per 100,000 person-years), and HS was 458.99 per 100,000 person-years (95%CI: 417.10–500.89). Table 3 lists the HR and their corresponding 95% CIs of future stroke after multivariable adjustment for each lipid variable. The incidence of all stroke increased by quartiles of TG (*P*_trend_ = 0.003), LDL (*P*_trend_ = 0.001), LDL/HDL (*P*_trend_ = 0.002), and TG/HDL (*P*_trend_ = 0.002). The incidence of IS increased by quartiles of TC, TG, LDL, non-HDL, TC/HDL-C, TG/HDL-C and LDL-C/HDL-C (all *P*_trend_ < 0.05) but not HDL (*P*_trend_ = 0.525). However, there was no association between lipid variables (quartiles) and future risk of hemorrhagic stroke.

**Table 3:** Adjusted^a^ Hazard Ratios (HRs) of Future Stroke between the participants According to quarter of baseline lipid levels

Lipid variables	Quarter 1	Quarter 2	Quarter 3	Quarter 4	*P*_trend_
TC (mmol/L)	< 4.52	4.52–5.17	5.17–5.84	≥5.84	
Ischemic stroke (HR,95% CI)	1.00	0.90 (0.64–1.28)	0.93 (0.66–1.32)	1.51 (1.10–2.07)	0.004
Hemorrhagic stroke(HR,95% CI)	1.00	0.63 (0.40–1.00)	1.11 (0.74–1.64)	0.73 (0.47–1.13)	0.492
All stroke (HR,95% CI)	1.00	0.79 (0.60–1.04)	1.02 (0.79–1.32)	1.16 (0.90–1.50)	0.067
TG (mmol/L)	< 0.93	0.93–1.32	1.32–1.98	≥1.98	
Ischemic stroke (HR,95% CI)	1.00	1.05 (0.74–1.49)	1.57 (1.13–2.18)	1.65 (1.18–2.31)	0.001
Intracerebral Hemorrhage(HR,95% CI)	1.00	1.26 (0.84–1.91)	1.12 (0.72–1.74)	1.24 (0.79–1.94)	0.548
All stroke (HR,95% CI)	1.00	1.11 (0.85–1.45)	1.35 (1.04–1.75)	1.48 (1.13–1.93)	0.003
HDL-C(mmol/L)	< 1.20	1.20–1.40	1.40–1.98	≥1.98	
Ischemic stroke (HR,95% CI)	1.00	0.91 (0.66–1.27)	1.02 (0.74–1.40)	1.07 (0.78–1.48)	0.496
Intracerebral Hemorrhage(HR,95% CI)	1.00	0.80 (0.52–1.23)	0.91 (0.60–1.37)	0.83 (0.55–1.27)	0.536
All stroke (HR,95% CI)	1.00	0.85 (0.66–1.11)	0.95 (0.74–1.23)	0.95 (0.74–1.23)	0.955
LDL-C(mmol/L)	< 2.29	2.29–2.73	2.73–3.22	≥3.22	
Ischemic stroke (HR,95% CI)	1.00	0.93 (0.65–1.33)	1.20 (0.85–1.69)	1.89 (1.36–2.61)	< 0.001
Intracerebral Hemorrhage(HR,95% CI)	1.00	1.22 (0.80–1.86)	1.18 (0.77–1.81)	1.96 (0.61–1.52)	0.774
All stroke (HR,95% CI)	1.00	1.04 (0.79–1.37)	1.18 (0.90–1.54)	1.48 (1.14–1.92)	0.001
Non-HDL (mmol/L)	< 3.23	3.23–3.76	3.76–4.29	≥4.29	
Ischemic stroke (HR,95% CI)	1.00	0.92 (0.65–1.30)	0.99 (0.70–1.39)	1.51 (1.09–2.09)	0.004
Intracerebral Hemorrhage(HR,95% CI)	1.00	0.76 (0.50–1.17)	0.93 (0.62–1.40)	0.68 (0.44–1.07)	0.169
All stroke (HR,95% CI)	1.00	0.85 (0.65–1.11)	0.98 (0.75–1.27)	1.13 (0.88–1.47)	0.167
TC/HDL-C	< 3.52	3.52–3.67	3.67–3.92	≥3.92	
Ischemic stroke (HR,95% CI)	1.00	1.91 (1.33–2.75)	1.43 (0.98–2.10)	2.02 (1.39–2.92)	0.002
Intracerebral Hemorrhage(HR,95% CI)	1.00	0.49 (0.32–0.75)	0.59 (0.39–0.89)	0.74 (0.49–1.10)	0.334
All stroke (HR,95% CI)	1.00	1.08 (0.83–1.40)	0.94 (0.72–1.23)	1.31 (1.01–1.70)	0.043
LDL/HDL-C	< 1.74	1.74–1.95	1.95–2.18	≥2.18	
Ischemic stroke (HR,95% CI)	1.00	1.11 (0.76–1.63)	1.85 (1.30–2.64)	2.06 (1.44–2.95)	< 0.001
Intracerebral Hemorrhage(HR,95% CI)	1.00	0.78 (0.52–1.15)	0.60 (0.39–0.92)	0.79 (0.51–1.20)	0.202
All stroke (HR,95% CI)	1.00	0.93 (0.71–1.22)	1.18 (0.90–1.53)	1.41 (1.08–1.84)	0.002
TG/HDL-C	< 0.67	0.67–0.94	0.94–1.44	≥1.44	
Ischemic stroke (HR,95% CI)	1.00	1.46 (1.05–2.02)	1.24 (0.87–1.76)	1.81 (1.29–2.55)	0.002
Intracerebral Hemorrhage(HR,95% CI)	1.00	1.03 (0.68–1.58)	1.18 (0.78–1.80)	1.22 (0.78–1.90)	0.333
All stroke (HR,95% CI)	1.00	1.27 (0.98–1.65)	1.20 (0.92–1.57)	1.58 (1.21–2.06)	0.002

^a^Included variables: age (years), sex, ethnicity, BMI, current smoking, heavy drinking, diabetes mellitus, SBP, DBP, and anti-hypertension drug treatment

*BP* blood pressure, *BMI* body mass index, *TC* Total cholesterol, *TG* Triglycerides, *HDL-C* high-density lipoprotein cholesterol, *LDL-C* low-density lipoprotein cholesterol

**Fig. 1:**
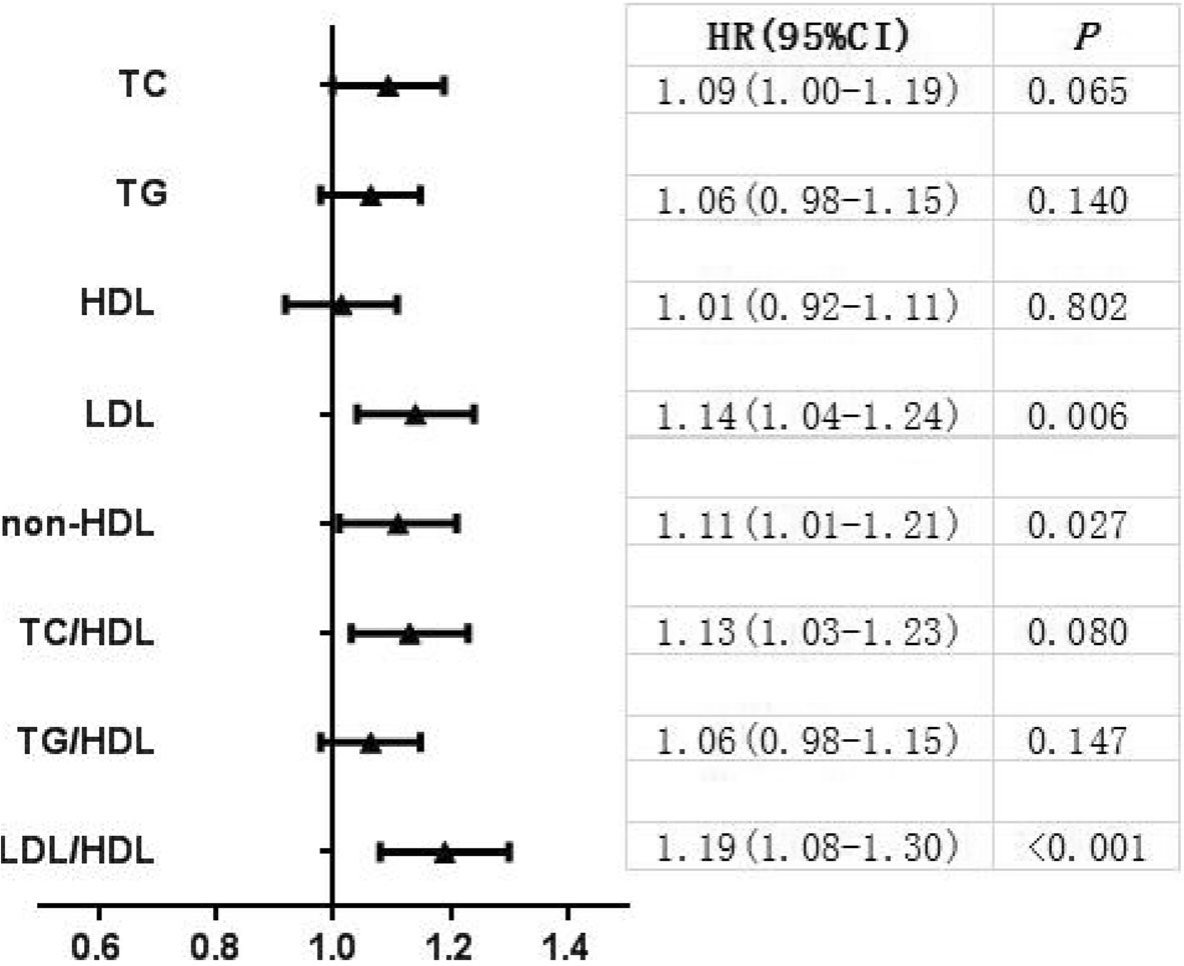
Adjusted hazard ratios and 95% CIs for future all stroke of each lipid variables according to 1-SD increase. Hazard ratios were adjusted for age, sex, ethnicity, BMI, current smoking, heavy drinking, diabetes mellitus, SBP, DBP, and anti-hypertensive medications

**Fig. 2:**
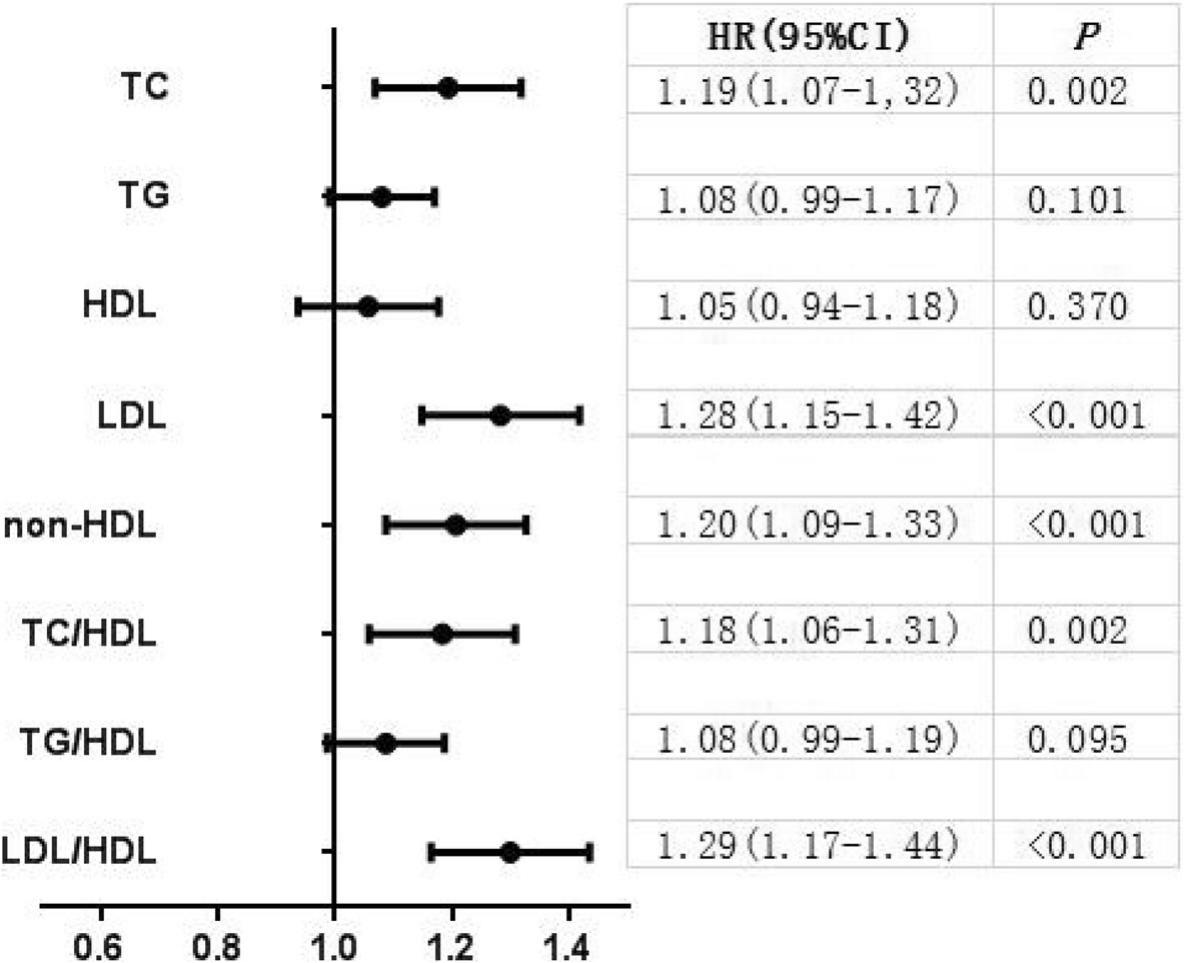
Adjusted hazard ratios and 95% confidence intervals for future ischemic stroke of each lipid variables according to 1-SD increase. Hazard ratios were adjusted for age, sex, ethnicity, BMI, current smoking, heavy drinking, diabetes mellitus, SBP, DBP, and anti-hypertensive medications

We further analyzed HRs for all stroke (Fig. 1) and IS (Fig. 2) associated with a 1-SD increase of all lipid variables. An increment of 1-SD in LDL, non-HDL, LDL/HDL was associated with greater HRs of 1.14 (95% CI: 1.04 to 1.24), 1.11 (95% CI: 1.01 to 1.21), and 1.19 (95% CI: 1.08 to 1.30) respectively for all stroke after multivariable adjustment. An increment of 1-SD in TC, LDL, non-HDL, TC/HDL, and LDL/HDL were associated with greater HRs of 1.19 (95% CI: 1.07 to 1.32), 1.28 (95% CI: 1.15 to 1.42), 1.20 (95% CI: 1.09 to 1.33), 1.18 (95% CI: 1.06 to 1.31), and 1.29 (95% CI: 1.17 to 1.44) respectively for IS after multivariable adjustment.

Table 4 shows the *P* value of differences in *β*-coefficients after standardization of traditional and non-traditional lipids. The HR was calculated by *β-*coefficient (*HR* = exp^(*β*)^). We could substitute the differences in HR values by comparing the *β-*coefficient differences. Therefore, we calculated the lipid variables’ corresponding *β* coefficients that had standardized and tested the differences of *β* between lipid variables according to the principle of correlation coefficient comparison. The order of *β* coefficient was: *β*_LDL/HDL_(0.252) > *β*_LDL_(0.237) > *β*_non-HDL_(0.172) > *β*_TC_(0.156) > *β*_TC/HDL_(0.153) > *β*_TG/HDL_(0.072) > *β*_TG_(0.071) > *β*_HDL_(0.044). The *β*_LDL/HDL_ had the biggest values in the non-traditional lipid variables and there was a marginally significant difference between *β*_LDL/HDL_ and *β*_LDL_ (*P* = 0.056).

**Table 4:** *P* values of differences in *β*-coefficients after standardization of traditional lipids and non-traditional lipids for ischemic stroke

Traditional lipids	Non-traditional lipids			
	_*β TG/*HDL_(0.068)	_*β* non-HDL_(0.095)	_*β TC/*HDL_(0.112)	_*β LDL/*HDL_(0.163)
_*β* HDL_(0.013)	0.005	< 0.001	< 0.001	< 0.001
_*β TG*_(0.057)	0.577	0.054	0.005	< 0.001
_*β TC*_(0.081)	0.509	0.476	0.114	< 0.001
_*β* LDL_(0.126)	0.003	0.113	0.474	0.056

## Discussion

Our study provided a comprehensive analysis of the association of lipoprotein particles (traditional lipids and non-traditional lipids) with risks of the incident all stroke, IS, and hemorrhagic stroke. We found that lipid levels were significantly associated with all stroke and IS (all lipid variables other than HDL were the risk factors), but the associations were attenuated for hemorrhagic stroke.

LDL-C was the important causal risk factors for CHD and IS which had been demonstrated in some observational studies or randomized controlled trials [28–31] and we also demonstrated the same relationship between LDL and IS. Our study observed the null associations differ from the previous studies that reported the inverse association of LDL-C with the risk of hemorrhagic stroke [32]. In the past studies, the associations of other lipids with stroke (all stroke, ischemic and hemorrhagic stroke) were not well established [28]. Bowman T S et al. [21] found TC, HDL, TG were not significantly associated with IS risk after adjustment with participants of Physicians’ Health Study (PHS). Zhang Y et al. [22] found TC had a significant association with hemorrhagic stroke in women. We found that all the lipids,by analyzing the relationship between traditional and non-traditional lipids and IS and hemorrhagic stroke risk, were negatively correlated with IS but not with hemorrhagic stroke. In addition to observational studies [14–20], randomized clinical trials have shown that lipid-lowering therapy can reduce the risk of IS in patients with previous IHD (ischemic heart disease) and this result was not validated in the non-IHD population [33]. At the same time, some clinical trials have found that lipid-lowering therapy can reduce the risk of IS [34, 35], but some studies have shown that it did not have the effect of reducing risk [18, 36, 37]. Another meta-analysis of 27 randomized trials about lipid-lowering therapy found a 21% reduction in IS risk per 1 mmol LDL-level reduction (95% CI, 15–26%). However, it was not known whether the beneficial effects of lipid-lowering therapy on IHD and IS were entirely due to a decrease in LDL concentrations or other lipid parameters such as TG [38–41]. That is, the composite application of lipid-related variables may have a stronger ability to predict disease.

The traditional lipids included TC, TG, LDL, HDL, and the non-traditional lipids included non-HDL, TC/HDL, TG/HDL, and LDL/HDL. In this study, we found that LDL, non-HDL, and LDL/HDL had association with the future all stroke status and TC, LDL, non-HDL, TC/HDL, and LDL/HDL had an association with the future IS status. According to the guideline, the main recommendation of clinicians for the development of lipid-lowering therapy was LDL [42–46]. From the comparison of the highest quartile and the lowest quartile, we found the HRs of the non-traditional lipids (TC/HDL, LDL/HDL) were higher than LDL for IS. And the HR of TG/HDL was higher than LDL for all stroke, although no statistical verification was performed (TG/HDL was considered to be a readily available marker of atherosclerosis and was associated with insulin resistance, CVD and all causes of death [5, 47]). Guo X et al. [48] found that non-traditional indicators were associated with an increased risk of IS in Chinese patients with hypertension by cross-sectional study, with the larger variables TG/HDL-C, LDL-C/HDL-C. Zhang Y et al. [22] found a positive association between TC/HDL-C and all stroke and IS but not hemorrhagic stroke. Another study [21] found the role of TC/HDL in the increased risk of IS was limited to those with the highest TC/HDL levels. However, none of the above studies compared the effect of traditional and non-traditional lipids on the risk of IS. So we carried on the traditional lipid variables and traditional lipid variables standardized *β*coefficient difference test. We found that the value of non-traditional *β*_LDL/HDL_ was the largest and higher than all traditional lipid variables. Except for *β*_LDL_, the difference between *β*_LDL/HDL_ and all the other traditional lipid variables were statistically significant.

Other studies suggested that the main cause of this heterogeneity in hemorrhagic stroke may not be driven by atherosclerosis, but more likely by high blood pressure and vascular fragility [31, 32, 49]. At the same time, the inefficacy of the individual and lipid variables in the hemorrhagic stroke may reflect the lack of statistical power to detect the real correlation between those in this study.

In order to better comprehend our result, some strengths and limitations must be mentioned. It is well known that the long follow-up time is crucial to determine the causal relationship between risk factors and some diseases. This study was a large-scale, prospective cohort study with a median follow-up time of 8.4 years. By comparing the statistic difference between the traditional and non-traditional lipids profiles, we found a better indicator, LDL-C/HDL-C. Given the predictive ability of it for stroke status, we should highly consider its application for hypertension patients as it may help to improve the efficacy of the individualized patient stroke risk assessment and guide clinical decisions. We failed to collect information on atrial fibrillation and antilipemic drug treatment, which would be possible confounders affecting the results of hypertension population. At the same time, the blood lipid measurement in this study was only carried out in a single time point, but the vascular damage in stroke was a dynamic and complicated process with time passing.

## Conclusion

This study found that, except for the traditional blood lipids in the hypertensive population, non-traditional lipids with a complex index also had a strong association with IS risk. This study did not find that any lipids variables were associated with the incidence of hemorrhagic stroke, which may be explained by the pathogenesis of lipids (mainly through atherosclerosis) [31]. Therefore, the lipids profiles of non-traditional variables should be considered for the daily management and prevention of IS in clinical practice.

## Abbreviations

BP: Blood pressure
CAD: Concluded angina pectoris, myocardial infarction, arrhythmia
CHD: Coronary heart disease
CI: Confidence interval
CVD: Cardiovascular disease
DBP: Diastolic blood pressure
GBD: Global Burden of Disease
HDL-C: High-density lipoprotein cholesterol
HR: Hazard ratio
ICH: Intracerebral hemorrhage stroke
IS: Ischemic stroke
LDL-C: Low-density lipoprotein cholesterol
SAH: Subarachnoid hemorrhage stroke
SBP: Systolic blood pressure
TC: Total cholesterol
TG: Triglycerides
TIA: Transient ischemic attacks
